# Evaluation of Apical Microleakage in Open Apex Teeth Using MTA Apical Plug in Different Sessions

**DOI:** 10.1155/2013/959813

**Published:** 2013-06-24

**Authors:** Mohammad Yazdizadeh, Zeinab Bouzarjomehri, Navid Khalighinejad, Leyli Sadri

**Affiliations:** ^1^Department of Endodontics, Ahvaz University of Medical Sciences, Ahvaz, Iran; ^2^Department of Pediatric Dentistry, Isfahan University of Medical Sciences, Isfahan 8174673461, Iran; ^3^Students Research Center, School of Dentistry, Isfahan University of Medical Sciences, Isfahan 8174673461, Iran

## Abstract

*Aim*. To compare microleakage of apexification using MTA in one or two sessions. *Materials and Methods*. 88 single rooted teeth were prepared and divided into two groups then received MTA apical plug. In the first group, the teeth were immersed in normal saline for 24 hours and then backfilled with guttapercha and AH26 sealer. In the second group, the teeth were obturated immediately after receiving apical plug. Four positive and four negative controls were selected. All specimens were placed in 1% methylene blue and decalcified in 5% nitric acid and finally were placed in methyl salicylate until getting transparent. All teeth were visualized for assessment of dye penetration under stereo dissecting microscope. *Results*. 36 and 35 teeth showed dye leakage in the first and second groups. Dye penetration into the entire canal length was confirmed in the positive control group, and in the negative control group no dye penetration was seen. Mean dye penetration in the first and second group was 5813 and 9152 **μ**m. *t*-test revealed a significant difference between dye penetrations of two groups (*P* < 0.05). *Conclusion.* MTA requires adequate time for setting in the presence of the moisture, and final obturation should be delayed until final setting of MTA.

## 1. Introduction

 The presence of vital pulp is essential for root development. Therefore, when the pulp is reversibly inflamed it is crucial to maintain pulp vitality [1]. Caries and traumas are the main cause of pulp necrosis. If these occur prior to root maturation, the root development would be halted, and it can lead to an open apex tooth [2].

 One of the main treatment methods of nonvital open apex teeth is apexification [3]. For many years calcium hydroxide (CaOH) has been the first choice of an intra canal dressing in apexification [4]; however, some drawbacks like coronal micro-leakage, tooth susceptibility to fracture [5], and multisession treatments [2, 6, 7] made clinicians look for an appropriate alternative for CaOH.

 It has been suggested that MTA plugs are more convenient and efficient compared to traditional CaOH [8]. MTA is used in different clinical cases in dentistry including direct pulp cap, internal resorption treatment, perforation management, and root canal filling [3]. Also MTA is used for pulpotomy in primary dentition [9–11]. One of the most interesting aspects of pulp-capping studies is utilizing this material for pulp treatment. It has been shown that MTA stimulates natural dentin repair at pulpal exposure sites during direct pulp cap [9]. It was declared that MTA can prevent the micro-leakage and stimulate the original tissue regeneration when it is in direct contact with pulp and periradicular tissues [8]. In some micro-leakage surveys MTA was shown to afford less micro-leakage than traditional materials [12, 13]. 

MTA powders consist of fine hydrophilic particles which form a colloidal gel in the presence of water or moisture [14]. In different studies, MTA has shown good sealing ability [15], acceptable marginal adaptation [16], and biocompatibility. MTA also can be used as an apical barrier [17].

The two session MTA apical barrier technique became increasingly popular among clinicians [18]. In this technique, a 5 mm MTA plug was used as an apical barrier, and then teeth were temporarily filled. In the next session, the teeth were evaluated, and root canals were filled [19]. However, because of patient compliance and occurrence of micro-leakage between sessions, Morse suggested the one-session apexification. In this method, the root filling is accomplished in the same session the MTA is used [7].

The importance of the coronal seal in preventing endodontic failure is well established. One session treatment method appears to be more convenient compared to common two sessions method. However, it is crucial to investigate the amount of apical micro-leakage in one and two session treatments to assess the superiority of one of these methods in preventing the apical micro-leakage. As a result, The present study was designed to compare these two treatment methods.

## 2. Materials and Methods

 This is a prospective experimental study. Eighty-eight freshly extracted, mature teeth with a single straight root were included. Teeth were stored in physiologic serum at the room temperature [20]. All specimens were intact and free of fracture, large restorations, or caries. Teeth were kept in sodium hypochlorite (0.5%) for 7 days to remove all debris and soft tissues.

### 2.1. Specimen Preparation

 Crowns were sectioned with a no. 577 bur in a high-speed handpiece perpendicular to the long axis of the teeth at a standard 15 mm measured from the apex. A no. 10 K-file was used to establish the working length. Working length was determined by measuring the length at which the no. 10 file was visible at the apical foramen and subtracting 0.5 mm. Canals were prepared initially using no. 15, 20, and 25 K-files sequentially. Then all canals were prepared by no. 2 and then no. 3 Gates Glidden drills in the measured working length. During instrumentation, canals were irrigated with 2 mL of 2.5% sodium hypochlorite (NaOCl) until irrigants exit from the apex. Final irrigation was accomplished using 1 mm normal saline to remove NaOCl remnants. Canals were dried by paper point. 

80 specimens were randomly divided to two experimental groups of 40 teeth. All specimens received 5 mm MTA barrier (ProRoot; Tulsa Dental, Tulsa, OK, USA). The density and the length of the plug were confirmed by radiographies. The MTA was packed again if the radiographies showed any void. Adding more MTA in these teeth reserved the thickness of 5 mm.

In the first group, 40 teeth were immersed in the normal saline until the MTA setting was completed. Then teeth were filled with gutta percha and AH26 sealer using reverse cone technique.

40 specimens in the second group were laterally filled with the same technique at the time of MTA plug insertion. Sealer remnants were removed by 99% alcohol.

Eight teeth were divided to two groups of positive and negative controls; each consists of 4 teeth. In positive control group, specimens were left unfilled, and in negative control group, canals were filled with gutta percha and AH26 using canal impression technique, and then the apex was sealed with sticky wax.

Then specimens' orifice was sealed using sticky wax after the remnants of the sealer, and gutta percha were removed.

Finally all specimens were incubated at 37°C in 100% humidity for 72 hours.

 After immersion in the normal saline at room temperature in order to dry the sealer, in all experimental and positive control groups, crown and root surfaces except for an area of 1 mm around the root apex were covered by two layers of nail varnish, and all crown and root surfaces were covered by one layer of nail varnish in negative control group. Then all teeth were immersed in 1% methylene blue for 72 hours. After one week all samples were washed with distilled water and excess ink and varnish were removed using no. 15 blade. Teeth were decalcified in 5% nitric acid then sequentially dehydrated in ethyl alcohol solutions with different concentrations (70%, 80%, 90%, and 100%) and finally immersed in methyl salicylate until getting transparent [21]. All teeth were examined under stereo dissecting microscope (Carl Zeiss, Oberkochen, Germany) to visualize dye penetration. 

*t*-test was used to show any statistically significant difference between experimental groups of the study. Statistical significance was defined at *P* < 0.05. 

## 3. Results

 Both experimental groups showed dye leakage. In the first group (24 hours setting of MTA) 4 teeth were excluded from the study due to apex resorption and lack of transparency. Also 5 teeth were excluded in the second group (canals were filled immediately after MTA placement) due to the same reason. As a result, 36 and 35 teeth showed dye leakage in the first and second groups, respectively. 

**Figure 1:**
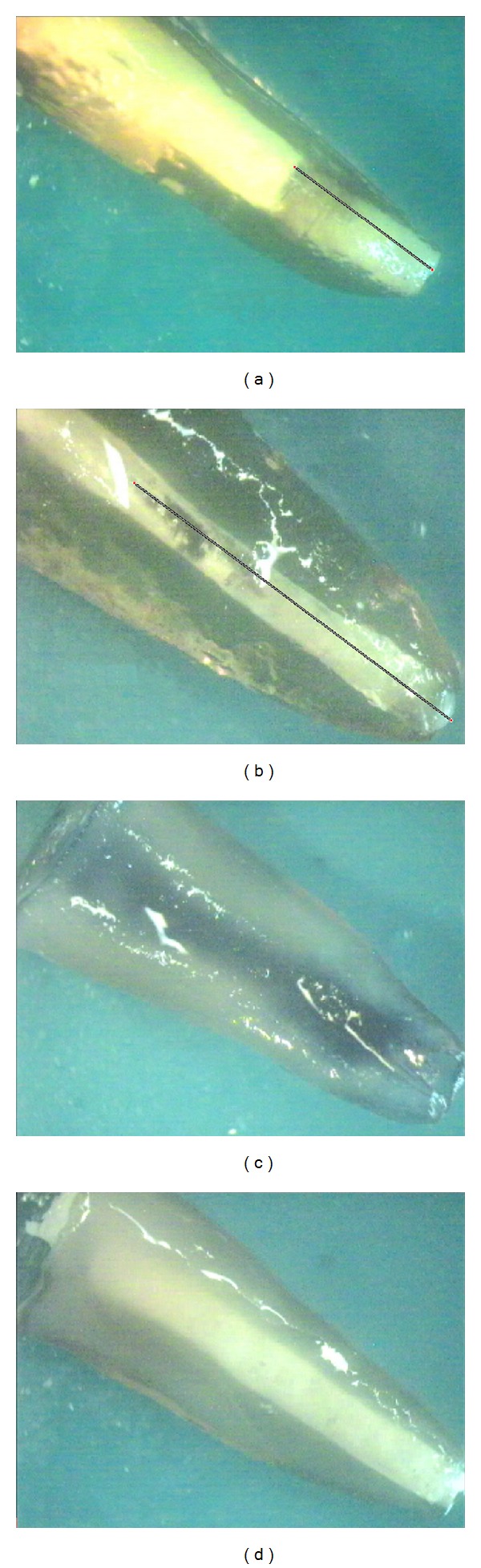
Dye penetration: (a) group 1, (b) group 2, (c) positive control, and (d) negative control.

Dye penetration into the entire canal length was confirmed in the positive control group. No dye penetration was observed in the negative control group. Dye penetrations in the experimental and control groups were shown in Figure 1.

Mean dye penetration in the first group (24 hours setting of MTA) and the second group (canals were filled immediately after MTA placement) was 5813 and 9152 *μ*m, respectively (Table 1).

*t*-test revealed that there was a significant difference between two groups regarding the mean amount of dye penetration (*P* = 0.01).

## 4. Discussion

 Various studies have declared that MTA provides an excellent apical seal, and MTA demonstrated its superiority over other commonly used materials [22–27]. The idea of single visit apexification is not new and has been examined for many years [28, 29].

 In the present study, the sealing ability of MTA was assessed under different conditions, and apical micro-leakage was measured using dye penetration length from apex in open apex canals filled with MTA. Since MTA requires 3-4 hours for appropriate setting in contact with moisture, the sealing ability of the MTA was assessed in two different times in the present study. 

 Methylene blue has been used in different studies to assess the micro-leakage [25, 26, 30]. In the present study, penetration length of 1% methylene-blue dye was also used. In the pilot study, which was conducted before experimental study, Indian ink was used. However, it did not show any penetration. The positive control group was used to assure the dye penetration, and also negative control specimens showed that apex is the only route of dye penetration. 

 The model used to instrument and create open apex teeth was in accordance with Pichardo study [31]. In the present study, 5 mm MTA apical barrier was used. This thickness was confirmed in Al-Kahtani et al. [32] and Lawley et al.'s [13] studies which showed that 5 and 4 mm MTA plugs respectively provide an absolute seal against micro-leakage. 

 In the present study, both groups (first group: 24 hours setting of MTA, second group: obturated immediately after MTA placement) showed micro-leakage. The results of the present study are in agreement with Vizgirda et al. [33]. It has been declared that leakage may be caused by the intracanal delivery technique (orthograde and retrograde) as orthograde delivery is more technique sensitive [34]. In the present study the MTA was delivered orthogradally. The results are contrary to the Al-Kahtani et al.'s [32] study which showed that 5 mm MTA can completely prevent bacterial micro-leakage. However, in Pichardo study [21] there was a sign of micro-leakage in MTA filled canals, despite that they were delivered retrogradally. It can be postulated that packing and adapting MTA to the dentinal walls play a more important role than MTA delivery technique. Also the micro-leakage has been assessed by a different technique like dye penetration or bacterial survey, and this may affect the results of the studies.

 Although a one-visit apexification procedure with MTA has been suggested, [30] the present study clearly revealed the superiority of two-step procedure over one-step procedure. In the present study, there was a significant difference in the amount of dye penetration between the experimental groups. MTA powder consists of hydrophilic particles that set in the presence of moisture. In the present study, specimens which were obturated 24 hours after MTA insertion showed the least dye penetration. 

 In the present study, MTA was used in both experimental groups, and it was not compared with other root end filling materials. However, MTA showed some degree of dye penetration. Micro-leakage in the present study can be attributed to the technique sensitive orthograde delivery of MTA or penetration ability of methylene-blue dye. Also doubt that remains in relation to the validity of results is the fact proven by Wu et al. [24] that MTA causes methylene-blue discoloration. Therefore, further studies should be conducted to use other micro-leakage models like radioisotopes, electro-chemical currents, and bacterial penetration. Furthermore, it seems rational to use retrograde models to assess its efficacy compared to orthograde filling.

## 5. Conclusion

 It can be concluded that MTA requires adequate time for setting in the presence of the moisture, and final obturation should be delayed until final MTA setting.

## Figures and Tables

**Table 1:** Mean, standard deviation, and maximum and minimum of dye penetration in two experimental groups in terms of micrometer.

Group	Number	Mean ± SD	Minimum leakage	Maximum leakage
1	36	5813 ± 1271	4010	9100
2	35	9152 ± 1913	7100	15205
